# North Central Asia isotopic database for archaeological samples

**DOI:** 10.1016/j.dib.2024.110032

**Published:** 2024-01-06

**Authors:** R. Smithers, A. R. Ventresca Miller, R. Fernandes

**Affiliations:** a Department of Archaeology, University of York, Kings Manor, York, UK; b Department of Anthropology, University of Michigan, Ann Arbor 48109, MI, United States; c Museum of Anthropological Archaeology, University of Michigan, Ann Arbor 48109, MI, United States; d Department of Archaeology, Max Planck Institute of Geoanthropology, Kahlaische Strasse 10, 07745 Jena, Germany; e Department of Bioarchaeology, Faculty of Archaeology, University of Warsaw, ul. Krakowskie Przedmieście 26/28, 00-927 Warszawa, Poland; f Masaryk University, Arne Faculty of Arts, Nováka 1, 602 00, Brno-střed, Czech Republic; g Princeton University, Climate Change and History Research Initiative, Princeton, USA

**Keywords:** Isotopes, Archaeology, Bioarchaeology, Central Asia, Collagen

## Abstract

The North Central Asia Isotopic Database (NCAID) is an open-access dataset of stable isotope measurements from archaeological remains, spanning from the Early Neolithic until present-day in North Central Asia. With 3,143 individual entries corresponding to data accumulated over more than 20 years of research, this comprehensive dataset encompasses measurements of stable carbon and nitrogen isotopes in organic fractions from archaeological humans, animals, and plants. NCAID incorporates diverse supporting information, providing geographical information, archaeological context descriptions, and chronology. This resource facilitates research into past human lifeways, paleo-environments/climates, and animal management practices throughout North Central Asia and will be continually updated as more novel data is released.

Specifications Table

**Table:** 

Subject	Earth and Planetary Sciences / Palaeontology
Specific subject area	Stable isotopes from archaeological samples provide valuable insights, including paleo-diets, paleo-mobility, paleo-environments/climate, and past animal management practices.
Data format	Secondary
Type of data	Table
Data collection	Data was collected from bibliographic research to assign to each sample an archaeological description, location, and chronology.
Data source location	Archaeological studies (references in text)
Data accessibility	Repository name: Pandora Data identification number: https://www.doi.org/10.48493/0g6y-6712 Direct URL to data: https://pandoradata.earth/organization/north-central-asia-isotopic-database
Related research article	Ventresca Miller, AR, Wilkin, S, Smithers, R, Larson, K, Spengler, R, Haruda, A, Kradin, N, Bazarov, B, Miyagashev, D, Odbaatar, T, Turbat, Ts, Zhambaltarova, E, Konovalov, P, Bayarsaikhan, J, Hein, A, Hommel, P, Nash, B, Nayak, A, Vanwezer, N, Miller, B, Fernandes, R, Boivin, N & Roberts, P (2023) ‘Adaptability of millets and landscapes: Ancient cultivation in North-Central Asia’. *Agronomy,* 13, 2848.

## 1 **Value of the Data**

• NCAID serves as a valuable research tool for archaeologists, physical anthropologists, and zooarchaeologists employing isotopic data.
• Paleo-isotopic data can be used to investigate ancient human dietary patterns, human and animal movements, and paleo-environments.
• NCAID can be used to identify spatial and temporal data gaps and propose new research avenues.

## 2 Background

North Central Asia holds profound historical and ecological importance. Historically, it served as a crucial segment of the Silk Road, fostering cultural and economic exchanges between East and West. The region was the cradle of nomadic empires like the Xiongnu or the Mongols. Ecologically, it boasts a rich biodiversity with unique flora and fauna adapted to its vast taiga and steppe ecoregions.

Isotopic research on archaeological materials from northern central Asia has been carried out since approximately two decades. Isotopic analysis of human, animal, and plant remains offer direct data on past human diets, human and animal mobility patterns, and paleo-environmental conditions. In the case of Central Asia, a historically pivotal region with legacies of nomadism, empire, and trade, isotopic studies have enriched our understanding of the complex interactions between the region's inhabitants and their environments over millennia. Isotopic research has complemented historical records and traditional archaeological findings, offering a more nuanced picture of the past in a region where written records can be sparse or absent for certain periods and locations.

## 3 Data Description

**Fig. 1:**
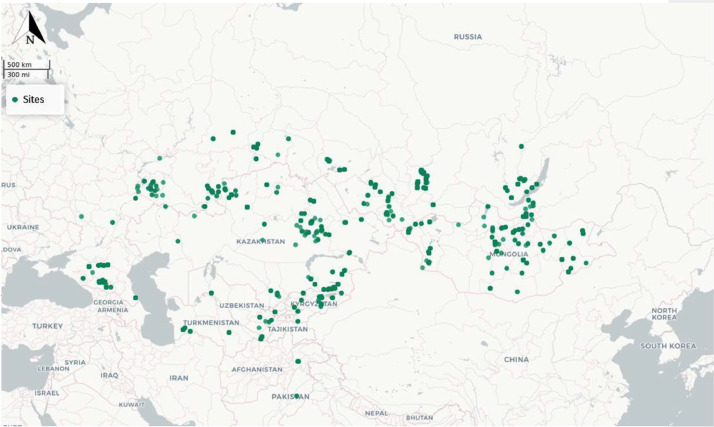
Site distribution across Mongolia, Russia, Kazakhstan, Kyrgyzstan, Pakistan, Tajikistan, Turkmenistan, and Uzbekistan.

The North Central Asia Isotopic Database (NCAID) consists of published stable isotope data for archaeological human, animal, and, plant samples from across North Central Asia (Fig. 1) [1], [2], [3], [4], [5], [6], [7], [8], [9], [10], [11], [12], [13], [14], [15], [16], [17], [18], [19], [20]. Data was retrieved from 35 of publications creating a dataset with 3,143 individual entries, consisting of 2,494 of stable carbon and nitrogen isotopic measurements. Sample chronology ranges from Early Neolithic to Modern samples. NCAID includes metadata on spatial location, archaeological context, sample description, chronology, and bibliographic references in addition to measurements of carbon and nitrogen stable isotopes on collagen, and carbon and oxygen stable isotopes on bioapatite. The North Central Asia isotopic database for archaeological samples is part of the IsoMemo network of autonomous isotopic databases.

The archaeological site is described across several fields: an archaeological project or general site name (Site information), the specific archaeological site name (Site name), a description of the site (Site description), information on the sample context (Context description), the original context identification (Context ID), the date of sample collection (Collection date), the archaeological culture associated with the sample (Archae. Culture), the site's altitude in meters (Altitude), and geographical coordinates in the WGS84 metric coordinate system—latitude (Latitude) and longitude (Longitude). Whether the coordinates were given in original publication or identified versus estimated (Exact Site location), the present-day country in which the site is located (Country), and its corresponding region (Region).

Information on samples is given across the following fields: the original publication sample ID (Sample ID), the original human or faunal individual ID (Individual ID), description of the sample (Sample description), taxonomic classification (Class, Order, Species), and the sample's common name (Common name). A broad category (General category) classifies the sample types into five main categories: animal, fish, food, human, and plant. Additionally, information about the diet (Diet) and domestication status (Domestication Status) of animals is also included. When available, details such as the individual's sex (Sex), age range (Age category individual), the minimum (Min. age individual) and maximum (Max. age individual) age range of a human, bone type from which a sample originates (Bone type), and from which part of a bone the same was taken (Bone part).

Two numeric fields are used to given the chronological range for each sample (Min Age (95 %) and Max Age (95 %)). The archaeological time period assigned to each sample (Period tags) is also given. Measurements of stable carbon (delta 13C coll) and nitrogen (delta 15N coll) isotopic values from the original. Further information, when present, on quality measurement indicators from the original source include collagen yield from the bone samples (Collagen yield), percentage of carbon (%C) and nitrogen (%N), carbon to nitrogen ration (C/N) are included.

Any key information absent from the original isotopic publication, such as archaeological culture, latitude, longitude, class, order, species, period tags, etc., was identified and reported using secondary sources. The bibliographic source, cited using Harvard style, complete with a journal URL (Link), DOI, and year of publication (Publication date), is provided for convenient reference to the original source material. If any absent information was extracted from a separate source this is listed as a secondary source (Secondary Reference Genetics/Radiocarbon).

## 4 Experimental Design, Materials and Methods

**Fig. 2:**
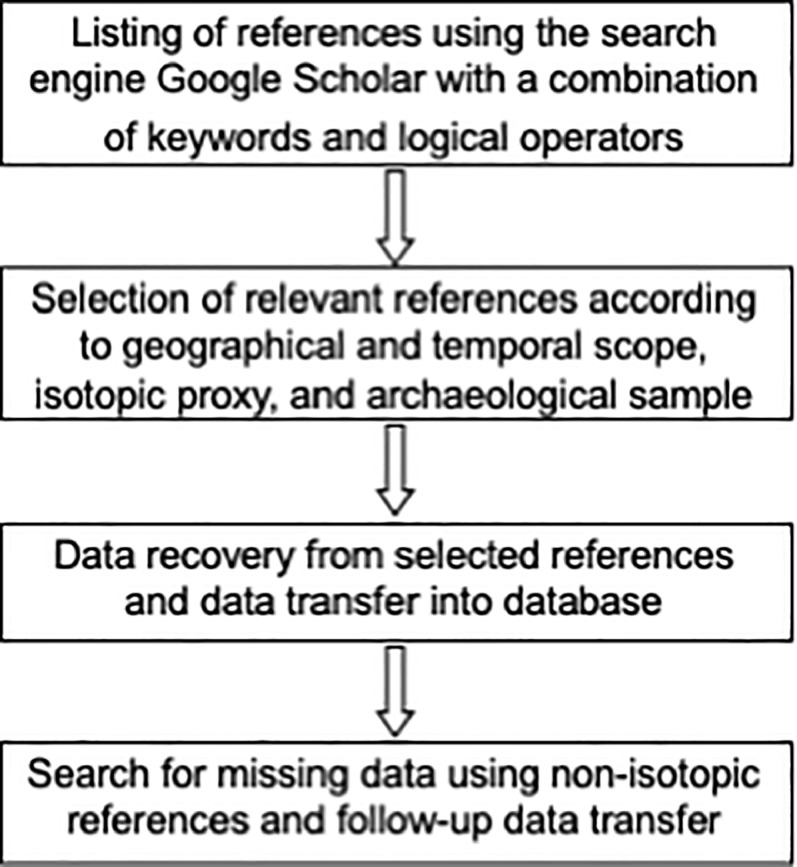
Data workflow.

Fig. 2 summarizes the data workflow employed in the compilation of the North Central Asia isotopic database for archaeological samples.

### 4.1 Search Strategy

The search engine Google Scholar was used to identify relevant data sources including scholarly articles and books which report archaeological stable isotope data for the study region. The search was conducted using key words such as ‘stable isotopes’ ‘carbon isotope’ ‘diet reconstruction’ ‘Central Asia’ and names of specific countries. Boolean operators (AND, OR) were employed to refine search queries. This search was performed up to July 2022.

### 4.2 Geographical and temporal scope

NCAID covers Mongolia, Russia, Kazakhstan, Kyrgyzstan, Pakistan, Tajikistan, Turkmenistan and Uzbekistan. The temporal range of isotopic values range from Early Neolithic to present-day. To the best of our knowledge, we identified all published studies related to stable isotopes in North Central Asia. Our focus was specifically on sites with carbon and nitrogen isotopic values to provide a synthesized dietary reconstruction of this region, aimed at determining the introduction of millet [1]. Additional information related to stable isotope collection (collagen yield, %C, %N, and atomic C/N) is included.

### 4.3 Secondary sources and metadata collection

Secondary genetic and radiocarbon sources were consulted to gather metadata, including site information, radiocarbon dates, longitude, and latitude. Various types of secondary sources, such as databases and published literature, were consulted for this purpose. Coordinates were obtained either directly from the original source or, in cases where precise coordinates were unavailable, by approximating the general site location within a few kilometers using the Google Maps mapping platform. The geographical coordinates are consistently reported in the WGS84 metric coordinate system.

### 4.4 Chronology and archaeological culture reporting

The chronology of the sample is presented as a temporal value in years BCE/CE, with a 95 % confidence interval represented by 'Min Age (95 %)' and 'Max Age (95 %)'. In instances where radiocarbon dates are unavailable, 'general dates' are approximated based on the site's archaeological culture context, considering factors such as material culture and radiocarbon dating methods. When specific information about the archaeological culture is missing, the culture is assigned based on the general time period and location. Archaeological culture assignments are primarily derived from the original publication. Additionally, 'Period Tags' are assigned to the radiocarbon dates to provide descriptive information about the time periods they represent.

### 4.5 Data reporting

A detailed description of the database metadata is included. The collected data, mentioned above, is reported in Excel, ODS, and CSV files. Metadata descriptors in Excel and ODS formats are also made available. All files can be retrieved via the Pandora data platform (https://pandoradata.earth/organization/north-central-asia-isotopic-database).

## Limitations

We compiled, to the best of our knowledge, all isotopic data from archaeological samples within the scope described previously. However, it is possible that some English references were missed and not included into the compilation. This will also be the case for references published in a language other than English. Whenever available we included standard quality criteria for the preservation status of bone collagen. However, these were not reported for all publications. Given the variety of cutoff values employed to select suitable collagen samples for isotopic analysis we did not classify data into preservation categories nor filtered out unsuitable samples. This must be done by data users prior to any data analysis. Our dataset also does not include all isotopic proxies employed in archaeological studies (e.g., strontium or sulfur isotopes are not included). These will be added in future versions of the compilation.

## Ethics Statement

This article meets the journal's Ethics and Policies requirements and does not involve animal or human studies.

## CRediT authorship contribution statement

**R. Smithers:** Conceptualization, Investigation, Data curation, Validation, Writing – original draft. **A. R. Ventresca Miller:** Investigation, Data curation, Writing – review & editing, Supervision. **R. Fernandes:** Visualization, Conceptualization, Writing – review & editing, Supervision.

## Data Availability

NCAID (Reference data) (Pandora).
